# Oxygen Transport during Ex Situ Machine Perfusion of Donor Livers Using Red Blood Cells or Artificial Oxygen Carriers

**DOI:** 10.3390/ijms22010235

**Published:** 2020-12-28

**Authors:** Silke B. Bodewes, Otto B. van Leeuwen, Adam M. Thorne, Bianca Lascaris, Rinse Ubbink, Ton Lisman, Diethard Monbaliu, Vincent E. De Meijer, Maarten W. N. Nijsten, Robert J. Porte

**Affiliations:** 1 Section of Hepatobiliary Surgery and Liver Transplantation, Department of Surgery, University of Groningen, University Medical Center Groningen, 9713 GZ Groningen, The Netherlands; s.b.bodewes@umcg.nl (S.B.B.); o.b.van.leeuwen@umcg.nl (O.B.v.L.); a.m.thorne@umcg.nl (A.M.T.); b.lascaris@umcg.nl (B.L.); v.e.de.meijer@umcg.nl (V.E.D.M.); 2 Surgical Research Laboratory, Department of Surgery, University of Groningen, University Medical Center Groningen, 9713 GZ Groningen, The Netherlands; j.a.lisman@umcg.nl; 3 Organ Preservation & Resuscitation Unit, University Medical Center Groningen, 9713 GZ Groningen, The Netherlands; r.ubbink@umcg.nl; 4 Department of Abdominal Transplantation Surgery and Coordination, University Hospitals Leuven, 3000 Leuven, Belgium; diethard.monbaliu@uzleuven.be; 5 Transplantation Research Group, Department of Microbiology, Immunology, and Transplantation, Katholieke Universiteit Leuven, 3000 Leuven, Belgium; 6 Department of Critical Care, University of Groningen, University Medical Center Groningen, 9713 GZ Groningen, The Netherlands; m.w.n.nijsten@umcg.nl

**Keywords:** machine perfusion, liver, gas transport, artificial oxygen carriers, oxygen, carbon dioxide, temperature

## Abstract

Oxygenated ex situ machine perfusion of donor livers is an alternative for static cold preservation that can be performed at temperatures from 0 °C to 37 °C. Organ metabolism depends on oxygen to produce adenosine triphosphate and temperatures below 37 °C reduce the metabolic rate and oxygen requirements. The transport and delivery of oxygen in machine perfusion are key determinants in preserving organ viability and cellular function. Oxygen delivery is more challenging than carbon dioxide removal, and oxygenation of the perfusion fluid is temperature dependent. The maximal oxygen content of water-based solutions is inversely related to the temperature, while cellular oxygen demand correlates positively with temperature. Machine perfusion above 20 °C will therefore require an oxygen carrier to enable sufficient oxygen delivery to the liver. Human red blood cells are the most physiological oxygen carriers. Alternative artificial oxygen transporters are hemoglobin-based oxygen carriers, perfluorocarbons, and an extracellular oxygen carrier derived from a marine invertebrate. We describe the principles of oxygen transport, delivery, and consumption in machine perfusion for donor livers using different oxygen carrier-based perfusion solutions and we discuss the properties, advantages, and disadvantages of these carriers and their use.

## 1. Introduction

Novel approaches are being explored to increase the number and quality of donor organs. These methods are aimed to decrease the donor organ shortages in relation to the number of candidate recipients on the waiting list [1]. One such approach is dynamic or machine preservation of donor organs. Machine perfusion (MP) is a procedure whereby organs are perfused ex situ after procurement. The aims of MP are multiple and include mitigation of ischemia-reperfusion injury (IRI), ability to prolong preservation time ex situ, improvement of organ condition and function, and assessment of organ viability prior to transplantation [2,3,4]. Currently, MP is widely studied and perfusion systems have been developed for various organs, including liver, heart, kidneys, and lungs [5].

MP of donor livers can be performed at different temperatures depending on the goal of the perfusion. For clinical use, temperature ranges are divided into three categories [6]. Hypothermic machine perfusion (HMP) ranges from 0 °C to 12 °C, at which the metabolic rate and energy expenditure of the organ is low. Oxygenated HMP mitigates IRI due to the resuscitation of mitochondria prior to warm reperfusion in the recipient. HMP can restore levels of intercellular adenosine triphosphate (ATP), reduce the production of reactive oxygen species (ROS), and confer protection to the bile ducts [7,8,9]. During HMP, organ viability and function cannot be assessed, because the liver is in a low metabolic state, but in addition it may prolong preservation time [10,11]. Subnormothermic machine perfusion (SNMP) ranges from 24 °C to 34 °C. During SNMP, at temperatures between 30 °C and 33 °C, the rate of metabolism increases to nearly 70% of the normal rate at body temperature. SNMP has the ability to increase ATP-levels and decrease mitochondrial injury and possibly asses viability [12,13]. In normothermic machine perfusion (NMP) organs are perfused at a temperature between 35 °C and 38 °C. During NMP, the metabolic rate is at normal physiological levels and the viability of the hepatocytes and cholangiocytes can be assessed [14,15,16]. A potential benefit of NMP is the use for therapeutic interventions [17]. NMP is technically more challenging, because the fully functioning liver has higher oxygen (O_2_) demands than during HMP. MP methods which are in experimental or preclinical evaluation such as subzero and hyperthermia conditions are beyond the scope of this review and are discussed elsewhere [18,19].

MP procedures are performed with different perfusion solutions depending on the temperature range. In HMP, an elementary perfusion solution contains the following ingredients: gluconate, phosphate, glutathione, allopurinol, and hydroxyethyl starch. Perfusion with this solution serves to provide O_2_ and remove waste products continuously produced by the liver [20]. In NMP, the perfusion solution should contain at least an oxygen carrier (OC), nutrients to maintain metabolism, anticoagulation, and broad-spectrum antibiotics [21]. To obtain the preferred osmolality and colloid osmotic pressure, water, saline, and human albumin or another type of colloid are usually added [21,22].

In this descriptive review we discuss the principles of gas transport during ex situ MP. Our focus is on the impact of temperature on gas transport and the various natural and artificial oxygen carriers (AOC) that can be added to the perfusion solution during MP from hypothermia to normothermia. We reviewed the published literature regarding MP of human livers and principles of gas transport. Experimental and clinical studies are discussed.

## 2. Overview of Gas Transport in Machine Perfusion

### 2.1. Physiological Gas Transport

In humans, gas exchange takes place by diffusion in the alveoli. The diffusing capacity of carbon dioxide (CO_2_) is 20 times higher than that of O_2_ [23]. After diffusion, 97% of O_2_ is absorbed by the red blood cells (RBC) where each hemoglobin (Hb) molecule binds four O_2_ molecules to iron ions (Fe^++^) to form oxyhemoglobin. The remaining O_2_ dissolves into the plasma. In the cell, O_2_ is consumed by the mitochondria in aerobic respiration to produce ATP [24]. In the citric acid cycle, the acetyl group of acetyl-coA is oxidized and produces CO_2_, which is transported back to the alveoli in a dissolved state (approximately 7%), in the form of bicarbonate (70%), or bound to Hb to form carbaminohemoglobin (20%) [25,26]. Thus, the transport of CO_2_ depends less on a carrier system than O_2_. Because CO_2_ also has a better diffusing capacity across membranes than O_2_, transport of CO_2_ is rarely a rate-limiting factor in MP.

#### 2.1.1. The Impact of Temperature on Gas Transport and Oxygen Requirements

**Figure 1:**
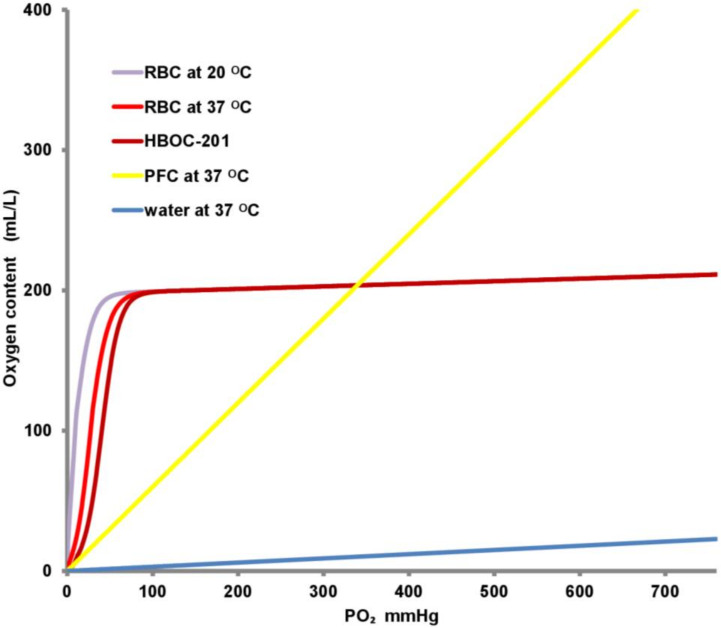
Graphical presentation of the relation between partial oxygen (PO_2_) pressure and the O_2_ content of different solutions. Note how poorly O_2_ dissolves in water (blue curve). To achieve a useful O_2_ content without an oxygen carrier, a saturation of 100% O_2_ is required, i.e., a partial pressure of 760 mm Hg. Perfluorocarbons (PFC; yellow curve) can dissolve 20 times more O_2_. The red blood cell (RBC) and hemoglobin-based oxygen carrier-201 (HBOC-201) curves assume a concentration equivalent with 7.76 mmol/L of O_2_ binding places [30]. Additionally, note that the only small apparent shifts in the dissociation curves result from the supraphysiological PO_2_ of 760 mmHg.

**Figure 2:**
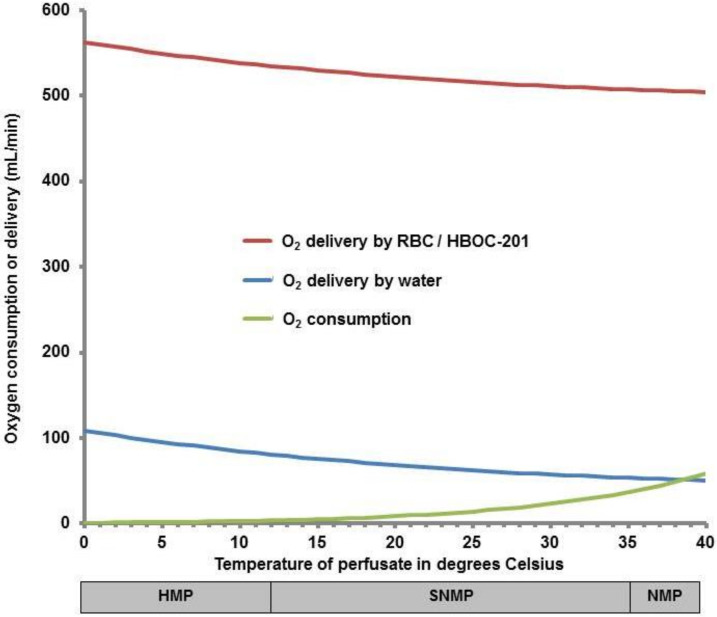
Graphical presentation of the relation between perfusion temperature and oxygen (O_2_) consumption (green curve) and O_2_ delivery. Metabolic rate and O_2_ consumption rise with approximately 10% for each increase in temperature measured in degrees Celsius. Note that the amount of O_2_ that is dissolved in water (blue curve) decreases at higher temperatures. At body temperature, O_2_ consumption/requirement will be larger than the amount that can be delivered by dissolved O_2_ alone, as indicated by the crossing green and blue curves. The addition of an oxygen carrier such as red blood cells (RBC) or hemoglobin-based oxygen carrier 201 (HBOC-201; red curve) can dramatically increase O_2_ content and delivery. Note that, as in whole body physiology, during organ perfusion oxygen delivery must be considerably higher than oxygen consumption, because oxygen consuming tissues such as the liver [31] can only extract a fraction of the delivered oxygen. The numbers displayed here assume oxygenation with 100% oxygen, a hemoglobin or HBOC-201 concentration equivalent to 7.76 mmol/L of O_2_-binding sites and a perfusion flow of 2300 mL/min.

Increasing blood temperature causes a right shift in the O_2_-Hb dissociation curve (Figure 1), supporting O_2_ release to the tissue. Increases in body temperature also lead to an increased metabolic rate, with higher O_2_ requirements. As is shown by the Van ‘t Hoff equation and the Arrhenius relation derived from it [27], the relation between metabolic rate and temperature is remarkably similar in various animal species, with an approximate 10% or 1.1-fold increase in metabolic rate per 1 °C (Figure 2) [28]. Likewise, increasing amounts of CO_2_ will be produced at higher temperatures [29]. 

When glucose is fully oxidized under steady-state conditions, the production of CO_2_ (V̇CO_2_) will be equal to the consumption of oxygen (V̇O_2_). The ratio between V̇CO_2_ and V̇O_2_ is called the respiratory quotient (RQ) and can be monitored in vivo or during ex situ MP. It is an important indicator of how ATP is generated. Glucose oxidation has an RQ of 1, whereas lipid oxidation has an RQ of <0.8 [23].

#### 2.1.2. The Impact of Temperature on Gas Solubility and Pressure in Fluids

Blood consists for approximately 92% of water and O_2_ dissolves poorly in water [32]. The solubility of a gas into a fluid is determined by Henry’s law: “At a constant temperature, the amount of a given gas dissolved in a given type of liquid is directly proportional to the partial pressure of that gas in equilibrium with that liquid.” This implies that a rise in the partial pressure of O_2_ (PO_2_) leads to proportionally more dissolved O_2_ in the fluid if the temperature is constant [33]. Delete blank row under the Equation (use backspace)

(1) Henry’s law: c=k· P

*c* = concentration dissolved gas, *k* = Henry’s law constant, *P* = partial pressure (in mm Hg or kPa).

However, when the temperature increases, O_2_ solubility decreases. Thus, at low temperatures, more O_2_ can be dissolved in the water compartment of a solution [32]. To maintain the same O_2_ concentration in a high-temperature solution without an OC in comparison to a low-temperature solution, the PO_2_ should be increased (see Figure 1 and Figure 2).

#### 2.1.3. Gas Transport in Machine Perfusion

During MP, O_2_ diffuses into the perfusate in the oxygenator, with a controlled gas flow and a set O_2_ fraction. The oxygenated perfusion solution enters the liver through the cannulated portal vein and, when dual perfusion is applied, also via the hepatic artery. The CO_2_ produced diffuses into the perfusate and circulates back to the oxygenator(s). The O_2_ content of the perfusate depends on the perfusate temperature, the administered O_2_ flow, and the concentration and use of either a cell-free solution or an OC-based solution.

Oxygenated HMP reduces IRI in contrast to non-oxygenated HMP or static cold storage (SCS) [7,16]. In hypothermic conditions the liver still maintains a low metabolic rate, therefore mitochondrial oxidative phosphorylation generates ATP when O_2_ is supplied. During oxygenated HMP the mitochondrial respiration decreases and ceases altogether after approximately 90 min [34]. At this point, the mitochondria have switched from a high-flux to a low-flux electron transfer stage. When O_2_ is not supplied to the solution, the mitochondria remain in the high-flux state electron transfer stage which causes the release of ROS during reperfusion [34,35,36]. 

The O_2_ requirements in HMP and SNMP can be met by adding dissolved O_2_ in the perfusion fluid [13,34]. In contrast, at 37 °C, the freely dissolved O_2_ concentration in blood is only 3%. As indicated in Figure 2, at higher temperatures, the O_2_ requirements exceed the maximal O_2_ delivery by dissolved O_2_ alone. It should also be noted, that the total delivery of O_2_ to any organ should be considerably larger than its consumption because of heterogeneity in O_2_ demand and metabolism. Consequently, a perfusion solution without an OC cannot supply sufficient O_2_ to the liver to maintain aerobic metabolism at normothermia [37,38]. 

In MP, oxygenation and CO_2_-removal are adjustable by controlling the gas flow and its O_2_ concentration (FiO_2_). The O_2_ and CO_2_ levels can be measured via arterial and venous blood gas analysis. In the arterial blood, the partial pressure of oxygen (aPO_2_), partial pressure of CO_2_ (aPCO_2_) and the percentage of Hb saturated with oxygen (asO_2_) can be measured. Likewise, the venous blood gas parameters vPO_2_, vPCO_2_ and vsO_2_ can be measured. Oxygen consumption can be reasonably accurately derived by subtracting the venous O_2_ content from the arterial O_2_ content.

(2) $$\mathrm{Oxygen}\;\mathrm{consumption}\;=\;V˙O_{2}\;\left(\mathrm{mL}/\min\right)\;=\;\mathrm{blood}\;\mathrm{flow}\;\cdot \;\left(1.36\cdot \mathrm{Hb}\cdot \left({\mathrm{asO}}_{2}-{\mathrm{vsO}}_{2}\right)\;+\;0.0031\cdot \left({\mathrm{aPO}}_{2}-{\mathrm{vPO}}_{2}\right)\right)$$

VO_2_ = oxygen consumption, bloodflow (L/min), Hb = hemoglobin (g/dL), sO_2_ = saturated oxygen (%), PO_2_ = perfusate partial pressure (mm Hg).

Similarly determining CO_2_ consumption is more challenging, as CO_2_ content is more difficult to accurately estimate in the arterial and venous samples.

## 3. Overview of Oxygen Carriers

**Figure 3:**
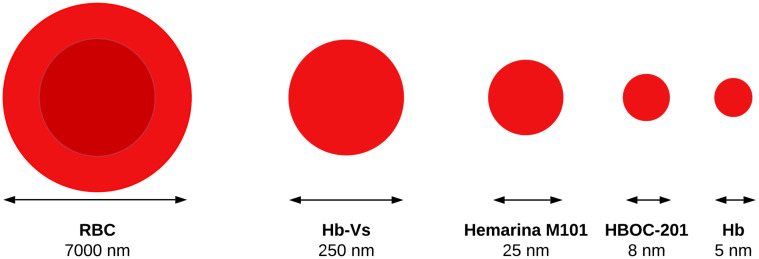
Visualizing the large differences in size between various oxygen carriers: red blood cells (RBC), hemoglobin vesicles (Hb-Vs), hemoglobin-based oxygen carrier 201 (HBOC-201), human hemoglobin (Hb).

As described above, ex situ MP of an isolated organ at normothermia requires the addition of an OC to the perfusion fluid to enable sufficient O_2_ delivery. Various OCs are currently used during machine preservation of donor organs. OCs have various characteristics resulting in different properties per carrier. In most studies on liver NMP, investigators have used RBCs as OC. Artificial alternatives are hemoglobin-based oxygen carriers (HBOCs) and natural extracellular OCs (Table 1, Figure 3).

### 3.1. Hemoglobin in Red Blood Cells

The human OC, Hb is transported by RBCs. Hb has a high affinity for O_2_ and a low affinity for CO_2_. One molecule of Hb can adsorb four molecules of O_2_. A higher concentration of Hb in the blood results in a higher capacity to transport O_2_. When an O_2_ molecule binds to Hb, the binding of additional O_2_ molecules is facilitated, which results in the S-shape of the O_2_-Hb dissociation curve (Figure 1). Human Hb further depends on 2,3-biphosphoglycerate, pH and temperature for its affinity for O_2_ [46].

It should be noted that when clinical laboratories report Hb concentrations in mmol/L, this concentration refers to the number of O_2_-binding sites and not to the actual number of Hb molecules. One molecule of Hb can bind to four O_2_ molecules and thus, in reality, a reported Hb of 10 mmol/L is only 2.5 mmol/L of Hb molecules. Accordingly, when Hb is expressed in mmol/L units, in order to calculate the total O_2_ content in a solution containing Hb, the amount of Hb should ***not*** be multiplied by 4 to arrive at the amount of O_2_ bound to Hb. This is, however, a common mistake in formulas that are used to calculate O_2_ consumption and it leads to an overestimation of O_2_ consumption by a factor 4. So, there is at least one advantage to the older formula that was based on the conventional unit g/dL: max O_2_ bound to Hb (mL/dL) = 1.36 Hb [47]. RBC-based perfusion solutions are only used in SNMP and NMP, due to hemolysis of the RBCs in a hypothermic environment. RBC are the most widely used OC in NMP [15,48,49,50]. Apart from Hb, RBCs also contain enzyme systems relevant for Hb protection and enhanced CO_2_ transport, such as methemoglobin-reductase and carbonic anhydrase. Some disadvantages of the use of RBCs are the relative scarcity of precious resources, immune-mediated phenomena, blood-borne infection transmission, and logistical difficulties with cross-matching. Considering that RBCs are a limited resource, alternative OCs have been examined for use in MP (Table 1) [51].

### 3.2. Hemoglobin-Based Oxygen Carrier

For decades, the development of AOCs has been a topic of extensive research. The first objective of AOCs was to substitute RBCs in blood transfusions to eliminate RBC-related side effects and to decrease the use of a scarce resource. Although the theoretical rationale for AOCs still exists, on account of the relatively short half-life and potential side effects when administered intravenously, the in vivo use of AOCs was never really successful. MP serves as a new niche for the application of AOCs because important systemic side effects are not relevant in MP. Clinical trials are currently using AOCs as possible substitutes for RBCs in the MP of single organs (Table 2) [52]. 

#### 3.2.1. Hemoglobin-Vesicles

Hemoglobin-vesicles (Hb-Vs) are phospholipid vesicles containing human-derived Hb. The diameter of the vesicles is 250–280 nm, which is smaller than that of RBCs. Hb-Vs are saturated for 50% at an O_2_ pressure between 9 mm Hg and 30 mm Hg. They do not contain clinically relevant RBC antigens and have a longer shelf life than RBCs. Although Hb-Vs are small in comparison to RBCs, they are large compared to macromolecules (Figure 3) and accordingly do not generate a colloid osmotic pressure. The half-life of Hb-Vs is two to three days, which limits their use [66,67]. A few studies have reported on the use of Hb-Vs in porcine and rat MP models for livers and limbs [63,68,69]. The porcine models showed increased O_2_ consumption during SNMP and decreased alanine aminotransferase and lactate dehydrogenase levels after reperfusion compared to HMP and SNMP, without an additional OC [62,63].

#### 3.2.2. Hemoglobin-Based Oxygen Carrier-201

The first-generation HBOCs prepared from modified tetramer Hb molecules, has been associated with vasoconstriction and renal dysfunction when administered intravenously. Clinical trials were suspended because of increased mortality, myocardial infarction, and stroke [70,71]. Hemoglobin-based oxygen carrier-201 (HBOC-201) can cause vasopressor effects due to a tetramer Hb, possibly through the binding of nitric oxide (NO) in the interstitial space, which leads to vasoconstriction and platelet aggregation. Additionally, HBOC-201 reduce the NO concentration due to its scavenging effects, which may explain the increased risk of myocardial infarction when administered intravenously [72,73]. However, many studies reevaluated the use of HBOC-201 and reported no evidence of NO-related toxic effects applicable to all HBOCs [74,75,76]. Later, clinical trials were performed using HBOC-201 in patients with severe anemia who could not receive whole blood products. Results indicated that patients with acute bleeding and hemolysis survived relatively longer than patients who did not receive HBOC-201 [77]. Currently, HBOC-201 is also used in US and European programs as a blood substitute for patients who do not accept transfusion with RBCs on account of their religious background [78]. In these compassionate use programs numerous patients have repeatedly received administration of HBOC-201 without clinical side effects (Z. Zafirelis, personal communication).

HBOC-201 or Hemopure^®^ (HbO2 Therapeutics LLC, Cambridge, MA, USA) is the most frequently used HBOC in MP. HBOC-201 is a polymerized Hb synthesized from bovine Hb. The Hb is extracted from bovine RBCs, purified, and cross-linked with glutaraldehyde to increase the stability and the molecular size. A purification process excludes possible harmful blood borne transmissions so that the end-product is a sterile pyrogen-free solution. These OCs are smaller than RBCs, resulting in a less viscous fluid. The affinity for O_2_ in HBOC-201 depends on the chloride ion concentration. It releases O_2_ easier than human Hb, because of a right shift in the Hb-dissociation curve (Figure 1). The in vivo half-life of HBOC-201 is approximately 20 h, which is much shorter than the half-life of RBCs, but sufficient for most cases of ex situ organ MP. The O_2_ pressure required for 50% saturation of the O_2_-binding sites in HBOC-201 is 38–40 mm Hg, which is higher than for human Hb in RBCs. When completely saturated, HBOC-201 can bind 1.36 mL O_2_ per gram of Hb. It has a molecular weight of 250 kDa [51,55,79]. A disadvantage of HBOC-201 is the formation of methemoglobin (metHb), which is formed when Fe^++^ is oxidized to Fe^+++^. Because the natural metHb-reductase in the RBCs that reduces Fe^+++^ back to Fe^++^ is absent, this leads to a gradual increase in the metHBOC-201 concentration in the perfusion fluid. MetHBOC-201 is longer available for O_2_ transport, leading to a lower saturation in prolonged machine preservation [80]. MetHBOC-201 can be converted back to normal, functional HBOC-201 by (repeated) addition of glutathione or ascorbic acid [79].

In several clinical studies HBOC-201 served as the OC in the MP of donor organs (Table 2). Laing et al. concluded that HBOC-201 could be used as an alternative for RBCs in NMP [51]. Van Leeuwen et al. used HBOC-201 in the DHOPE-COR-NMP trial, rewarming discarded human donor livers from hypothermic to normothermic conditions. In this trial suboptimal livers were continuously perfused and oxygenated, which increased the utilization of initially discarded donor organs. An advantage of HBOC-201 is that it can be used during the HMP phase which avoids the need to change the perfusion fluid when one switches from hypothermia to normothermia [55,57,60]. After MP, the perfused organ is flushed, leaving only minimal amounts of HBOC-201 to reach the recipient. Apart from visceral organ MP, Vrselja et al. used HBOC-201 to perfuse porcine brains to study if brain circulation and cellular functions could be restored (Table 2) [59].

### 3.3. Natural Extracellular Oxygen Carrier Hemarina M101

The natural extracellular Hb equivalent Hemarina M101 (HEMO2life^®^, France) is obtained from a marine invertebrate: *Arenicola marina*, a lugworm. The molecule is quite large with a molecular weight of 3600 kDa. It is composed of 156 globins and 44 non-globin linker chains that can carry up to 156 O_2_ molecules when saturated, which results in a high O_2_-binding capacity. Hemarina M101 is active over a large range of temperatures (4 °C to 37 °C) and releases O_2_ according to a simple gradient that does not require any allosteric effector. The molecule possesses intrinsic Cu/Zn-superoxide dismutase antioxidant activity that, to a certain extent, protects tissue from superoxide radicals [81].

Hemarina M101 was first described as a new and potentially promising blood substitute in 1997. The initial transplantation-related research was performed by Thuillier et al. They demonstrated that Hemarina M101 has a beneficial effect during SCS before kidney transplantation by decreasing chronic fibrosis and organ dysfunction [82]. Alix et al. compared SCS solution with Hemarina M101 versus SCS solution alone during porcine liver graft preservation, showing higher ATP levels in the SCS with Hemarina M101 group after 80 min [42,83]. M101 was also used in preclinical trials as an additive to the perfusion solution during HMP in a marginal kidney porcine model. A reduction of short-term function loss and no loss of function or tissue integrity were observed (Table 1) [43,84].

### 3.4. Perfluorocarbons

Perfluorocarbons (PFCs) are hydrocarbons in which practically all hydrogen atoms are replaced by fluoride. They have a high capacity for dissolving respiratory gasses and have an O_2_ solubility that is 20 times higher than that of water. As in the case of water, the amount of O_2_ that can be dissolved in PFCs is determined by Henry’s law. A high partial O_2_ pressure is necessary to maximize O_2_ content of the PFCs (Figure 1). The O_2_ dissociation curve of PFCs is a straight line in contrast to the sigmoid curve of Hb in RBCs. For the intravascular use of PFCs, the lipophilic molecule should be formulated as an emulsion, which inevitably will reduce its overall O_2_ content. PFCs have been used in static cold preservation with active and non-active oxygenation [85,86]. In 1994 PFCs were already used in a pulsatile subnormothermic setting for the preservation canine kidneys [64]. Recently, PFCs were used during porcine ex vivo lung perfusion, which showed better preservation of mitochondrial function, glucose consumption and neutrophil infiltration [65].

## 4. Future Perspectives

The majority of OCs used in clinical MP of donor organs are RBCs. HBOC-201 may be a promising alternative to RBCs in MP. The ideal perfusion solution should contain a cell-free OC that can be used for MP at all temperatures, and it should have a long shelf life. Future research should aim to elucidate the relation between metabolic rate and required O_2_ delivery in donor organs.

## 5. Summary

MP is a dynamic organ preservation platform technology used to increase the number and quality of donor organs. It can be performed at different temperatures of the perfusate solution–varying from hypothermic to normothermic perfusion. Low temperatures lead to a decreased metabolic rate, while at 37 °C the organ is metabolically active at a physiological level.

At normothermia, oxidative phosphorylation is required to generate sufficient ATP. In MP, O_2_ is added to the perfusion solution through the oxygenators, while CO_2_ diffuses out passively through the oxygenators. The O_2_ requirements in HMP and SNMP can be met with dissolved O_2_ in the perfusion fluid. Because O_2_ solubility decreases with increasing temperatures, in NMP a perfusion solution containing an OC is required.

## Figures and Tables

**Table 1:** Overview of the advantages and disadvantages of different oxygen carriers.

Oxygen Carrier	Advantages	Disadvantages	Pharmacokinetics	Possible Toxicity in Humans
Hemoglobin in red blood cells (RBC)	Within its physiological microenvironment Human blood product Low methemoglobin production Dynamic shift of O_2_-hemoglobin dissociation curve	Immune-mediated phenomena Blood-borne infection transmission RBC hemolysis during hypothermic machine perfusion (HMP) Cross- matching difficulties Precious resource	T ½ = 115 days MW = 64 kDa [Hb] = 12–15 g/dL P50 = 27 mm Hg	ABO incompatibility
Hemoglobin-based oxygen carrier-201 [39]	Easy O_2_ release to tissue Sterile and pyrogen-free Large temperature range (4 °C–37 °C) Less viscous than RBC Long shelf life: three years Compatible with all blood types	Formation of methemoglobin Short half-life Systemic vasoconstriction Lower O_2_ affinity than RBC	T ½ = 20 h MW = ~250 kDa [Hb] = 13 g/dL P50 = 38–40 mm Hg	Systemic vasoconstriction
Hemoglobin vesicles [40,41]	Absence of RBC antigens Smaller than RBC sDo not generate colloid osmotic pressure Do not rupture Long shelf life: two years	Only used in animal models	T ½ = 2–3 days [Hb] 10 g/dL P50 = 9 mm Hg	Release of free Hb can cause renal toxicity
Hemarina M101 [42,43]	Preliminary evidence in static cold storage Large temperature range (4 °C–37 °C) Simple gradient release O_2_ High O_2_ affinity Non-immunogenic	Only used in preclinical HMP and clinically in static cold storage	MW = 3600 kDa P50 = 7 mm Hg	None reported
Perfluorocarbons [44,45]	High O_2_ solubility Inexpensive Obey Henry’s law O_2_ uptake and release insensitive to environment	Formulated as emulsion, which reduces O_2_ content Needs high PO_2_ to maximize O_2_ content	T ½ = 8–24 h Emulsion dependent	Visual loss
Water	Delivers sufficient O_2_ below 20 °C Inexpensive	O_2_ content decreases with higher temperature leading to a mismatch above 20 °C	T ½ = ∞ MW = 18 Da	None

MW = molecular weight, T ½ = half-life, [Hb] = concentration of Hb or its equivalent for other carriers, P_50_ = O_2_ tension where 50% of Hb is saturated with O_2._

**Table 2:** Overview of ex situ (sub)normothermic machine perfusion using artificial oxygen carriers.

Author and Year	Oxygen Carrier	Temperature Range	Machine	Sample Size n	Transplanted n	Species and Organ
Fontes et al. [53] AJT 2015	HBOC-201	SNMP	Liver Assist	6	6	Porcine livers
Sadowsky et al. [54] Front Pharmacol 2016	HBOC-201	SNMP	Liver Assist	6	6	Porcine livers
Matton et al. [55] Liver Transpl 2018	HBOC-201	NMP	Liver Assist	24	-	Human livers
Laing et al. [51] Transplantation 2017	HBOC-201	NMP	Liver Assist	5	-	Human livers
Boteon et al. [56] AJT 2018	HBOC-201	NMP	Liver Assist	10	-	Human livers
De Vries et al. [57] AJT 2019	HBOC-201	HMP to NMP	Liver Assist	7	5	Human livers
Aburawi et al. [58] AJT 2019	HBOC-201	NMP	Kidney Assist	7	-	Human kidneys
Vrselja et al. [59] Nature 2019	HBOC-201	NMP	-	32	-	Porcine brains
Van Leeuwen et al. [60] Ann Surg 2019	HBOC-201	HMP to NMP	Liver Assist	16	11	Human livers
Bhattacharjee et al. [61] Transplantation 2020	HBOC-201	SNMP	-	5	-	Porcine kidneys
Shonaka et al. [62] Transplantation 2018	Hb-Vs	SNMP	-	3	-	Porcine livers
Shonaka et al. [63] Plos one 2019	Hb-Vs	SNMP	-	5	-	Porcine livers
Brasile et al. [64] Biotechnol. 1994	PFC	SNMP	-	4	-	Canine kidney
Inci et al. [65] Cells 2020	PFC	SNMP	-	11	4	Porcine lungs

## Data Availability

Data sharing not applicable.

## Abbreviations

AOC	artificial oxygen carrier
aPCO_2_	arterial partial pressure of carbon dioxide
aPO_2_	arterial partial pressure of oxygen
ATP	adenosine triphosphate
asO_2_	arterial percentage of Hb saturated with oxygen
CO_2_	carbon dioxide
Fe^++^	iron ion
FiO_2_	fraction of inspired oxygen
Hb	hemoglobin
HBOC	hemoglobin-based oxygen carrier
HBOC-201	hemoglobin-based oxygen carrier-201
Hb-Vs	hemoglobin-vesicles
HMP	hypothermic machine perfusion
IRI	Ischemia reperfusion injury
metHb	methemoglobin
MP	machine perfusion
NMP	normothermic machine perfusion
NO	nitric oxide
O_2_	oxygen
OC	oxygen carrier
PCO_2_	partial pressure of carbon dioxide
PFCs	perfluorocarbons
PO_2_	partial pressure of oxygen
RBC	red blood cell
ROS	reactive oxygen species
RQ	respiratory quotient
SCS	Static cold storage
SNMP	subnormothermic machine perfusion
SO_2_	oxygen saturation
VCO_2_	production of CO_2_
VO_2_	consumption of O_2_
vPCO_2_	venous partial pressure of carbon dioxide
vPO_2_	venous partial pressure of oxygen
vsO_2_	venous percentage of Hb saturated with oxygen
